# Direct Measurement of Surfactant-Mediated Picoforces among Nanoparticles in a Quasi-Two-Dimensional Environment

**DOI:** 10.1021/acs.langmuir.2c01928

**Published:** 2022-09-29

**Authors:** Roberta Ruffino, Nunzio Tuccitto, Gianfranco Sfuncia, Giuseppe Nicotra, Giovanni Li-Destri, Giovanni Marletta

**Affiliations:** † Laboratory for Molecular Surfaces and Nanotechnology (LAMSUN) and CSGI, Department of Chemical Sciences, University of Catania, viale A. Doria 6, 95125 Catania, Italy; ‡ Consiglio Nazionale delle Ricerche, Istituto per la Microelettronica e Microsistemi, 95121 Catania I, Italy

## Abstract

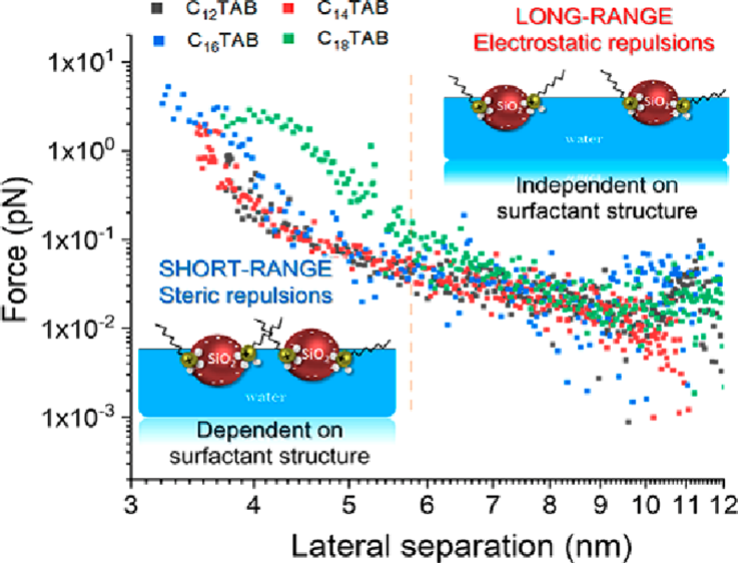

The lack of methodologies which enable us to measure forces acting between nanomaterials is one of the factors limiting the full comprehension of their behavior and their more effective exploitation in new devices. Here we exploit the irreversible adsorption of surfactant-decorated nanoparticles at the air/water interface to investigate interparticle forces and the effect of the surfactant structure on them. We measured the interparticle repulsive forces as a function of the modulation of the interparticle distance by simultaneously performing compression isotherms and the grazing incidence small-angle X-ray scattering (GISAXS) structural characterization of the monolayers at water–vapor interfaces. Our results demonstrate that the short-range interparticle forces are strongly affected by the presence of the organic ligands, which are shown to be able to influence the interparticle repulsions even when added in micromolar amounts. In particular, we demonstrate the predominant steric nature of short-range forces, which are accounted for in terms of the compression-induced stretched-to-coiled conformational transition of the ligand hydrophobic tail.

## Introduction

It is essential to understand and control interactions between nanoparticles (NPs) and measure nanoscale forces for the effective application of nanomaterials and nanomaterial-based devices in various fields spanning the design of smart materials^1−3^ to nanomedicine.^4,5^ Starting from the first pioneering works exploiting interactions between bare nanoparticles,^6^ it was shown that the complexity and the potential uses of nanoscale and interparticle forces can be enormously increased via the addition of proper ligands and molecules.^7−10^ This perspective, on the one hand, has stimulated the interest in theoretical and experimental tools contributing to the full depiction of the energy landscape of nanoscale interactions^11^ and, on the other hand, has highlighted the lack of a theory for the reliable prediction of the stability of sub-50-nm colloidal particles. Indeed, the Derjaguin–Landau–Verwey–Overbeek (DLVO) theory does not satisfactorily model the interactions between nanoscale objects, as its main approximation, e.g., the nonadditivity of the various interaction potentials, cannot be applied when interacting particles and molecules forming the propagation medium have comparable sizes.^12^ Furthermore, from the experimental point of view, optical tweezers, which have successfully been employed for the determination of interparticle forces between micrometer- and submicrometer-sized particles,^13−15^ cannot be applied to nanoparticles. It is possible to perform qualitative investigations of interparticle forces between NPs irreversibly adsorbed at liquid interfaces by compressing the so-obtained NP monolayer.^16^ Moreover, by simultaneously recording the compression work and the associated interparticle distance reduction via synchrotron radiation grazing incidence X-ray small-angle scattering (GISAXS),^17^ it is possible to measure, with subnanometer resolution, the interparticle forces as an average over a large number of NPs. In this framework, negatively charged silica nanoparticles assembled at liquid interfaces upon decoration of oppositely charged surfactants^18−20^ are a particularly suitable model system for the investigation of ligand-mediated interparticle forces. Indeed, owing to the availability of various cationic surfactants having the same charged head but different hydrophobic tail lengths, this system might allow us to investigate the effect of the surfactant tails on the strength and distance range of interparticle repulsions. In particular, when these experiments are performed on uniform sparse silica NP monolayers, obtained by adding minute amounts of cationic surfactants,^17^ they might allow us to probe the interparticle forces within a broad range of interparticle distances. We also stress that, given the large separation between interfacial NPs, these monolayers are expected to be more sensitive to subtle variations in the interparticle forces induced by different environmental changes. Finally, the comprehension of forces acting between nanoparticles trapped at liquid interfaces is of paramount importance for the development of tailored low-dimensional structures, as liquid surfaces and interfaces provide a promising environment for the spontaneous assembly of molecules and nanomaterials into 2D or quasi-2D systems.^21−30^ In fact, the finite contact angle at the nanoscale between NPs and the liquid subphase^31^ leads to the formation of three distinct interfaces, namely, the NP/subphase, NP/second fluid, and subphase/second fluid. Thus, NPs assembled at liquid interfaces represent a model system for the investigation of forces simultaneously propagating across two different media.^32^

Based on the above, the present study is mainly aimed at investigating and measuring the repulsions acting between negatively charged silica NPs assembled at the air/water interface upon the addition of micromolar concentrations of surfactants having the same cationic head (trimethylammonium bromide, TAB) but different hydrophobic tail lengths (ranging between C12 and C18). Our results, which show for the first time how interfacial repulsive forces scale with the interparticle distance and surfactant tail length, provide new experimental evidence for the origin of both short- and long-range repulsions acting between nanoparticles and propagating across quasi-2D asymmetric environments.

## Materials and Methods

A 34 wt % aqueous dispersion of negatively charged silica nanoparticles (Ludox TMA), having a nominal density of 2.1 g/cm^3^, was purchased from Sigma-Aldrich (Milan, Italy) and dialyzed to remove impurities via a dialysis tube (Membra-Cel MC18 with a molecular weight cutoff of 14 000 Da), purchased from Sigma-Aldrich (Milan, Italy), and then placed in a 400 mL beaker filled with ultrapure water under constant agitation. The nanoparticle average diameter, as determined by transmission electron microscopy (TEM), is 24.6 ± 3.9 nm (Supporting Information Figure S1).

NaCl and surfactants having the same trimethylammonium cationic head and different tail lengths, namely, dodecyltrimethylammonium bromide (C_12_TAB), myristyltrimethylammonium bromide (C_14_TAB), hexadecyltrimethylammonium bromide (C_16_TAB), and octadecyltrimethylammonium bromide (C_18_TAB), were purchased from Sigma-Aldrich (Milan, Italy) and used as received.

Silica/C_*n*_TAB dispersions were prepared by mixing the proper volumes of C_*n*_TAB solution and silica dispersions and by subsequent dilution to obtain the desired concentration. For the addition of NPs to the surfactant solutions that caused flocculation, surfactant solutions were duly diluted before adding NPs. This allowed us to avoid any unwanted flocculation at intermediate (higher) NP or surfactant concentrations. All of the dispersions discussed here are stable, as no flocculation occurred during their preparation. All of the dispersions investigated here had a constant NP concentration (0.1% wt), and the C_*n*_TAB concentration varied between 1 × 10^–7^ and 1.5 × 10^–2^ M. NaCl (1 mM) was also added to the dispersions to promote the surfactant adsorption onto nanoparticles.^33^ Then, the dispersions were immersed in an ultrasonic bath for 30 min to promote homogenization.

TEM measurements were performed with a JEOL ARM200F Cs-corrected microscope equipped with a cold-field emission gun with an energy spread of 0.3 eV and operating at 200 keV. Micrographs were acquired in conventional TEM (CTEM) mode using a Gatan UltraScan 1000XP (2*k* × 2*k*) charge-coupled device camera.

The surface tension values and the compression isotherms were recorded with a KSV Minitrough (Helsinki, Finland) equipped with a paper Wilhelmy plate for measuring the surface pressure (Π) as
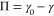
1where γ_0_ is the surface tension of pure water (72.8 mN/m at 20 °C) and γ is the surface tension of the solution/dispersion after the adsorption of the monolayer. For “static” surface tension measurements, solutions and dispersions were poured into a glass Petri dish immediately after the ultrasonic treatment, and then the surface tension was measured for 1800 s. Although previous studies suggest that longer times are required to reach equilibrium,^34^ this time was sufficient to record almost constant surface-pressure values. For compression experiments, the monolayer compression modulus was calculated as
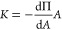
2where *A* is the macroscopic area value at which the tangent of the compression isotherm was measured.

Grazing incidence small-angle X-ray scattering (GISAXS) experiments were conducted at the Sirius beamline of the Soleil Synchrotron (Paris, France) with an 8 keV monochromatic X-ray beam having an incident angle of 0.11°, e.g., 92% of the water/air critical angle for total external reflection, with respect to the water surface. The GISAXS data were recorded with a Pilatus 1 M 2D detector placed at a distance of 2.517 m from the sample. GISAXS “static” measurements without compression were performed in a 10-cm-diameter circular Teflon trough, while GISAXS experiments during compression were performed in a Langmuir trough mounted in the beamline to simultaneously record GISAXS data and the compression isotherm.

GISAXS measurements were performed, in the case of “static” conditions, via the integration of 10 consecutive 2D signals recorded for 1 s while, during compression in the Langmuir trough, the GISAXS data recorded for 1 s were used without any further integration. By adopting these conditions we did not observe any radiation damage, as shown in Figure S2 of the Supporting Information. For data analysis, the GISAXS data were converted to 2D patterns of the intensity of the scattered X-rays as a function of the two components of the wave vector transfer *q*_*z*_ and *q*_*xy*_,^35^ where *q*_*z*_ is the wave vector transfer component perpendicular to the surface^35^
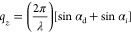
3and *q*_*xy*_ is the wave vector transfer component parallel to the surface^35^
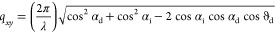
4where λ is the wavelength of the X-ray beam, α_i_ is the incident angle of the X-ray beam, α_d_ is the out-of-plane scattering angle, and θ_d_ is the in-plane scattering angle.

Peak fitting was performed on 1D graphs obtained by cutting and integrating the GISAXS pattern along *q*_*z*_ over the range 0.01 Å^–1^ ≤ *q*_*z*_ ≤ 0.02 Å^–1^. The peaks at positive and negative *q*_*xy*_ were independently fitted with the following equation:
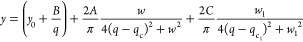
5The first term represents the background, the second term is the Lorentzian peak resulting from the integration of the Bragg rod centered at *q*_c_, having an area *A* and a full width at half-maximum (fwhm) *w*. The third term is the eventual second weaker peak centered at √3*q*_c_ ≤ *q*_c1_ ≤ 2*q*_c_, having an area *A*_1_ and a fwhm *w*_1_.

## Results and Discussion

**Figure 1:**
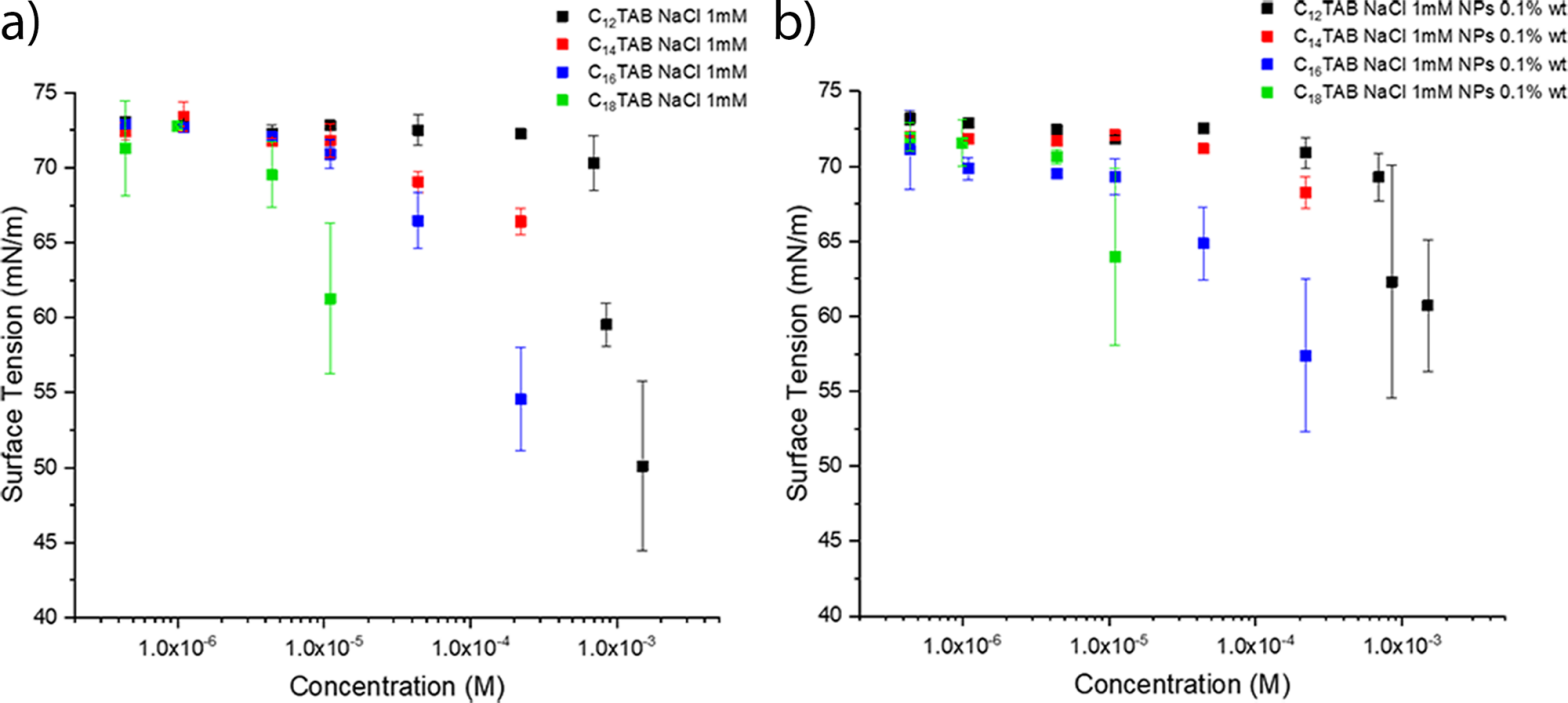
Variation of the surface tension as a function of the different structures and concentrations of surfactants. (a) Surface tension of solutions containing only NaCl and surfactants. (b) Data referring to dispersions containing, in addition to surfactants and NaCl, silica NPs at 0.1 wt % concentration. In both cases, the NaCl concentration is 1 mM.

The measurement of the interparticle repulsions by compression of the NP monolayers requires the avoidance of any interference caused by the adsorption of free surfactant molecules. Since it was previously reported that, above a threshold surfactant/NP bulk ratio, free surfactants predominantly adsorb at the expenses of NP/surfactant complexes,^17^ we preliminarily performed a systematic characterization of the surface tension and structure under static conditions of mixed surfactant/NP dispersions at various bulk surfactant concentrations. In Figure 1, the surface tension of the various systems studied here is reported as a function of the surfactant concentration for surfactants alone and for surfactant/NP dispersions. In both cases, the behavior is dominated by the surfactant adsorption at the water–air interface, yielding, as expected, a decrease in the surface tension with concentration. Given the higher surface activity of surfactants with longer tails, the bulk surfactant concentrations required to reduce the surface tension decrease with the tail length. The trend does not markedly change when mixed surfactant/NP dispersions are investigated. This confirms that NPs in themselves do not significantly contribute to the surface tension reduction^16^ and that, in turn, the surface tension is not diagnostic of the NP adsorption although it must be recalled that the surface tension values of mixed NP/surfactant dispersions were recorded at times shorter than the ones required for complete equilibration.^34^ It must also be mentioned that, by increasing the surfactant tail length, the surface tension measurement was possible for smaller concentration ranges, owing to the reduced solubility of surfactants with longer tails.

**Figure 2:**
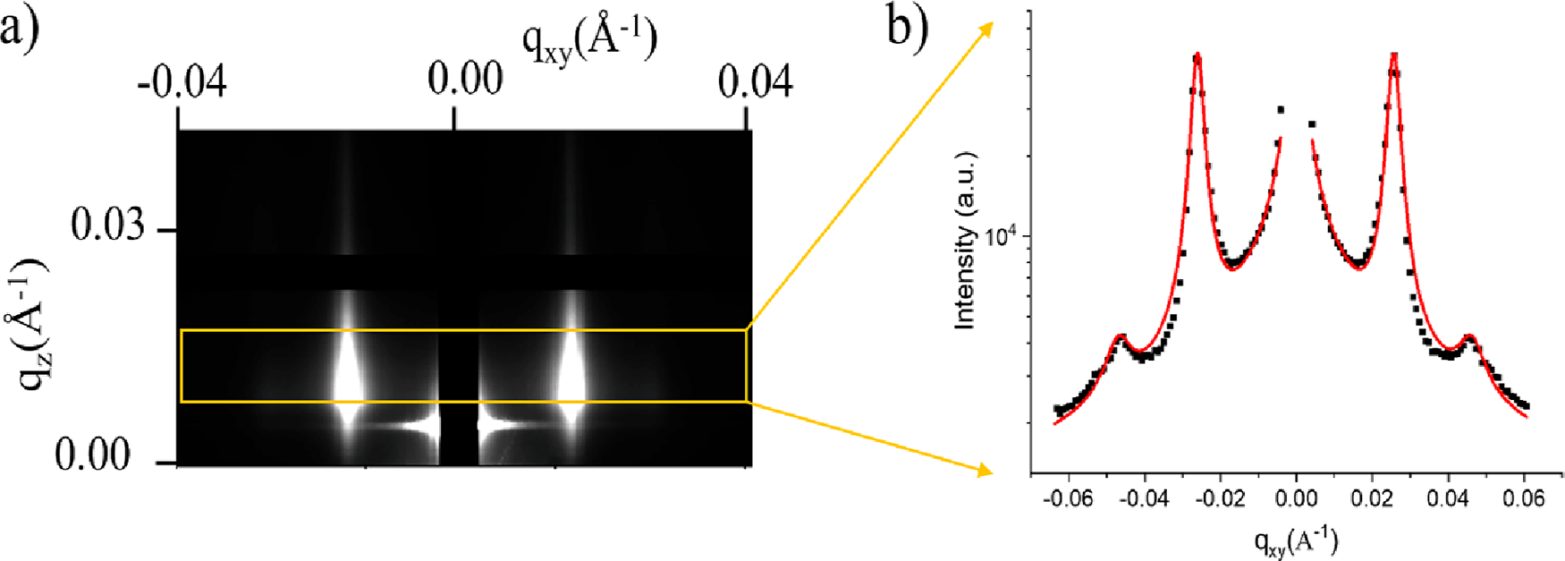
(a) Example 2D GISAXS pattern recorded from a silica NP monolayer adsorbed at the air/water interface. The two intense Bragg rods, in white, are associated with the average periodic distance between adjacent nanoparticles. By performing an integration for 0.01 ≤ *q*_*z*_ ≤ 0.02 Å^–1^ (yellow box), the 1D graph in (b) is obtained. Fitting of the 1D graph (details in Materials and Methods) led to the interparticle distance determination.

Cationic surfactants are known to promote the formation of homogeneous micro and NP monolayers even at very small bulk concentrations.^36,37^ Indeed, also for values of the dispersion surface tension which are basically equal to that of pure water, GISAXS measurements on these dispersions already reveal the presence of an NP homogeneous monolayer. This is clearly evident from the occurrence of two symmetric Bragg rods, which are the structure factors of the GISAXS pattern (Figure 2a). These rods, which are absent for simple “pure” surfactant solutions and “pure” NP dispersions (without surfactants),^17^ originate from the interference of X-rays scattered by NPs adsorbed at the interface and depend on the average interparticle distance (*D*). Thus, upon fitting of the GISAXS horizontal cuts (Figure 2a,b, Materials and Methods, and Supporting Information Figures S3–S6), *D* can in turn be evaluated from the equation
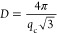
6where *q*_c_ is the fitted peak position. The above equation considers a local 6-fold coordination^38^ which gives rise to the intense (10) peak at *q*_c_ and to a fainter peak placed between *q*_c_ and 2*q*_c_, which are the positions of the (11) and (20) peaks, respectively, as seen in the horizontal cut along *q*_*z*_ for 0.01 ≤ *q*_*z*_ ≤ 0.02 Å^–1^ (Figure 2b).

**Figure 3:**
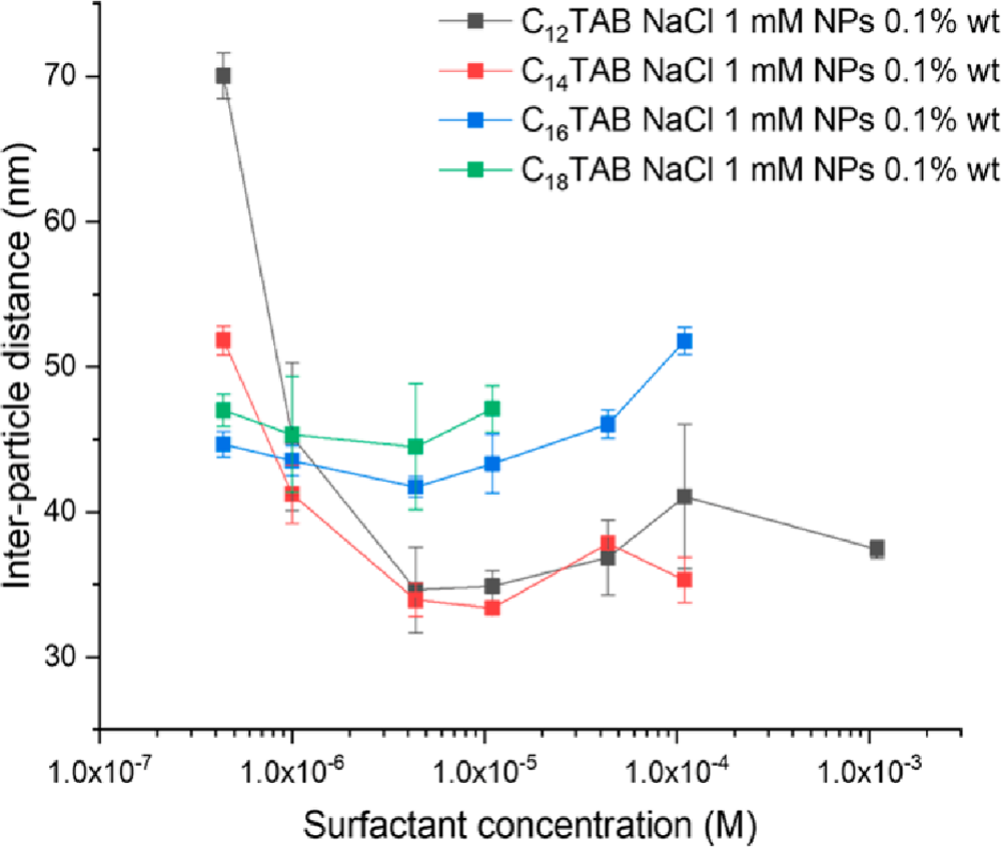
Interparticle distance measured as a function of C_*n*_TAB concentration with 0.1 wt % NPs and 1 mM NaCl. At micromolar concentrations, the gradual increase in the C_*n*_TAB concentration involves a reduction in the interparticle distance for C_12_TAB and C_14_TAB, while for surfactants having longer hydrophobic tails, the interparticle distance is mostly unaffected by the surfactant concentration.

Figure 3 shows that the nanoparticle adsorption is substantially dependent on both the surfactant nature and concentration. Note, however, that the interparticle distances obtained from the reported GISAXS experiments may not correspond to the equilibrium ones. Indeed, it has been reported that, for more concentrated NP solutions (SiO_2_ 1%), the maximum coverage is reached at longer times (i.e., after 10^4^ s or more^34^) than the one allowed in the present experimental protocol. The measured interparticle distance (i.e., the monolayer density) is adjustable over a range of 40 nm for C_12_TAB by modulating the concentrations between 4.4 × 10^–7^ and 4.4 × 10^–6^ M. This broad range is slightly reduced for C_14_TAB, while for surfactants having longer hydrophobic tails (i.e., C_16_TAB and C_18_TAB), the interparticle distance is mostly unaffected by the concentration and, at the lowest surfactant concentration investigated here, is lower than the one measured for shorter surfactants. We believe that this is due to the increasing surface activity with tail length which prompts the adsorption of more NPs. Previous studies conducted on monolayers formed upon addition of C_16_TAB to silica Sicastar NPs, which were not synthesized and provided by the same supplier of Ludox NPs investigated here, showed significant variations of the interparticle distance with surfactant concentration.^17^ The difference may arise either from the different NP size or from other differences between silica NPs provided by different suppliers. However, both systems showed the lowest interparticle distance at 4.4 × 10^–6^ M C_16_TAB.

Remarkably, the monolayer density is comparable, for all four surfactants, at a surfactant bulk concentration of 1.1 × 10^–6^ M (Figure 3). The same observation was previously reported by Walker and co-workers^16^ by using 10^2^ higher concentrations for both nanoparticles and surfactant. This suggests that the NP interfacial behavior may be similar over a broad range of bulk concentrations, provided that the bulk surfactant/NP ratio is kept constant.

Above 4.4 × 10^–6^ M, further increases in the surfactant concentration for both long and short tails do not strongly affect the interparticle distance, which remains substantially constant or even slightly increases. Concerning this last point, we recall the work of Maestro et al., showing that for C_16_TAB/NPs complexes, above a certain threshold concentration, the surfactant forms double layers on the aqueous side of the NPs.^31^ Such double layers may explain the observed increase in the interparticle distance. However, in our systems, the lowest lateral separations between adjacent NPs range between ∼8 and ∼20 nm, well above the expected bilayer thickness.^39^ Therefore, we argue that, in the systems investigated in this work, sparse NP monolayers always form, and above 4.4 × 10^–6^ M, both surfactant-decorated NPs and free surfactants adsorb at the air/water interface.^17^ This effect could also justify the shorter interparticle distance measured for shorter-tailed surfactants at bulk concentrations higher than 10^–6^ M. In particular, given their higher surface activity, longer-tailed surfactants might more effectively adsorb at the air/water interface and, in turn, they might more effectively hinder the NP adsorption. Indeed, surfactant adsorption^40^ or surfactant-induced NP interfacial displacements^41^ were already reported when increasing the sodium dodecyl sulfate concentration in the presence of silica NPs adsorbed at the water/hydrocarbon interface.

In order to compare the effect of the surfactant tail length on the interparticle forces, NP monolayers having similar densities, i.e., NPs having comparable surface activities, and the smallest possible amount of adsorbed free surfactant are needed. Therefore, we decided to focus our investigation on two bulk surfactant concentrations, namely, 1.1 × 10^–6^ and 4.4 × 10^–6^ M.

**Figure 4:**
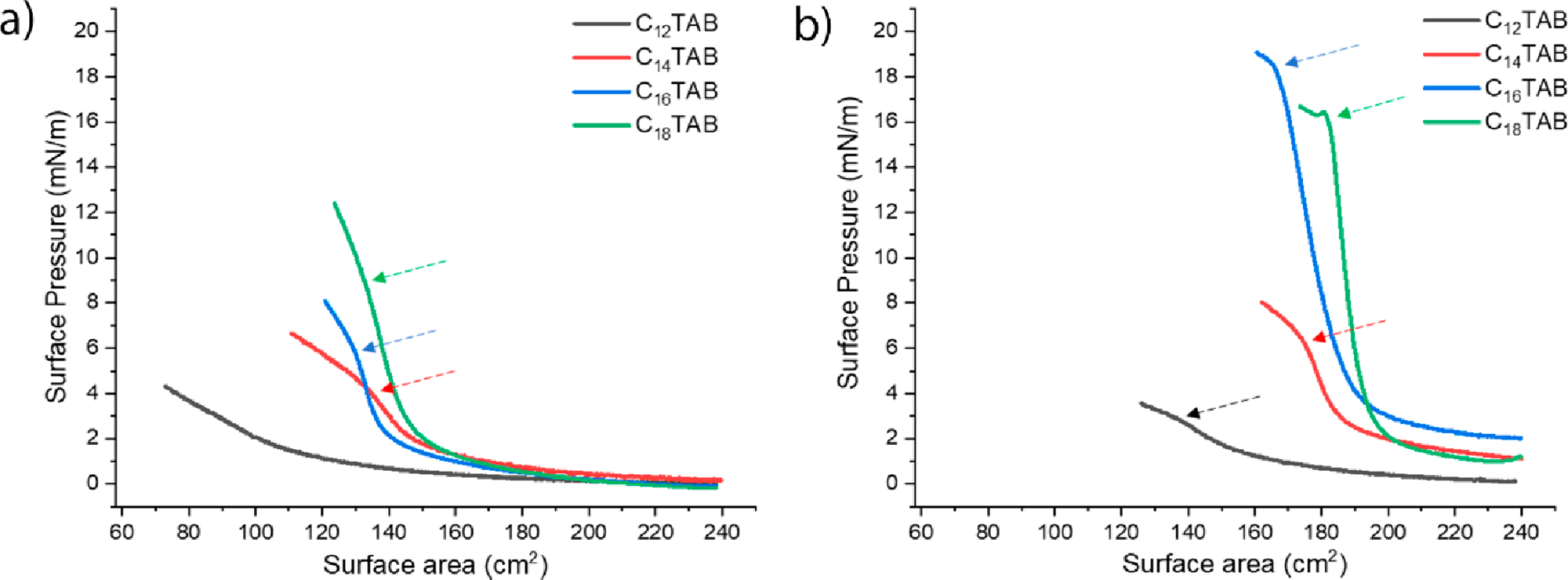
Compression isotherms recorded for dispersions having 1 mM NaCl, 0.1 wt % NPs, and surfactant concentrations of (a) 1.1 × 10^–6^ and (b) 4.4 × 10^–6^ M. As the hydrophobic tail length and the surfactant concentration increase, the monolayer becomes less compressible, showing a rapid increase in the surface pressure for larger surface areas. The arrows indicate the limiting compression rate. (See the structural characterization below for further details.)

The compression isotherms of mixed NP/surfactant dispersions having surfactant concentrations of 1.1 × 10^–6^ and 4.4 × 10^–6^ M are reported in Figure 4. Note that, notwithstanding at these concentrations the measured surface tension is substantially equal to that of pure water (∼72.8 mN/m, Figure 1), the compression isotherms show remarkable surface-pressure variations following the monolayer compression but only in the presence of NPs. Indeed, the solution with surfactant alone (Supporting Information Figures S7 and S8) shows no or small variations in surface pressure with compression.

This behavior can be explained in terms of the desorption of the molecules from the monolayer at the air/water interface. In fact, the adsorption of surfactant molecules is a reversible equilibrium since the desorption energy of the surfactant is lower than *k*_B_*T*, causing the free surfactant molecules to desorb from the compressed interface. This leads, for C_12_TAB-C_16_TAB, to constant surface pressure with compression while C_18_TAB shows slight surface-pressure increases with compression probably caused by the slower desorption kinetics (Supporting Information Figures S7 and S8). At variance with this, the adsorption of the NPs/C_*n*_TAB complexes at the air–water interface is irreversible under the conditions of the experiments, as far as the desorption energy is greater than *k*_B_*T*.^42^ Therefore, although we cannot unambiguously prove, even at micromolar surfactant bulk concentrations, the absence of adsorbed free surfactant molecules, Figures S7 and S8 demonstrate how, in the micromolar regime, these molecules mostly desorb with compression. In other words, the compression-induced surface-pressure increase is due to the gradual approach of NPs, “modulated” by the repulsive interparticle forces opposing this approach.^16,17^

**Figure 5:**
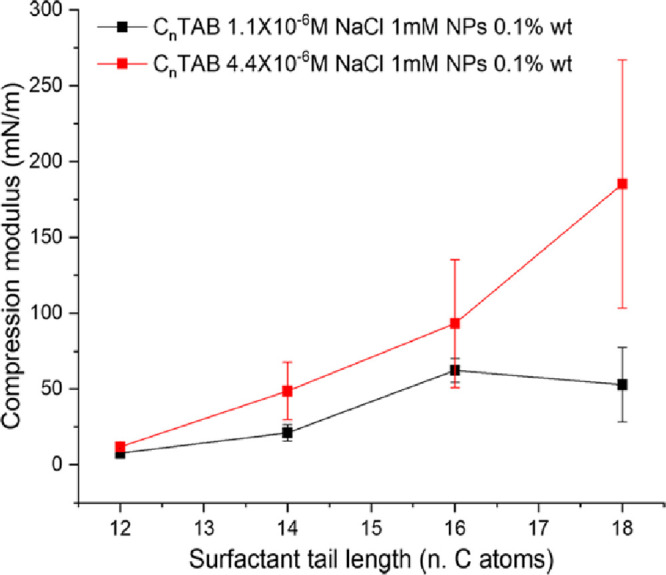
Compression moduli as a function of the surfactant tail length at surfactant concentrations of 1.1 × 10^–6^ M (black squares) and 4.4 × 10^–6^ M (red squares).

In this context, the monolayer compression modulus could be evaluated from the compression isotherms, showing a clear dependence upon both the surfactant tail length and concentration (Figure 5). In particular, monolayers formed by surfactants with longer hydrophobic tails show both higher moduli and a higher sensitivity to surfactant concentration.

Contrary to what was observed under static conditions, where the monolayer density at 1.1 × 10^–6^ M is not significantly affected by the surfactant tail length (Figure 3), micromolar amounts of surfactants with different structures markedly influence the monolayer compressibility. This suggests that the repulsive interactions acting during the monolayer compression depend on the surfactant structure and that, in particular, they presumably consist of steric contributions. Similarly, the moduli recorded at 4.4 × 10^–6^ M agree with this hypothesis, as surfactants with longer hydrophobic tails lead to higher moduli, despite the lower monolayer density (Figure 3).

Overall, the compression experiments prove that, in the micromolar regime investigated here, short-range forces have a predominant steric origin. This supports the idea that, even at low surfactant/NP bulk ratios, surfactants directly attach to nanoparticles adsorbed at the air/water interface and influence the short-range interparticle interactions.

**Figure 6:**
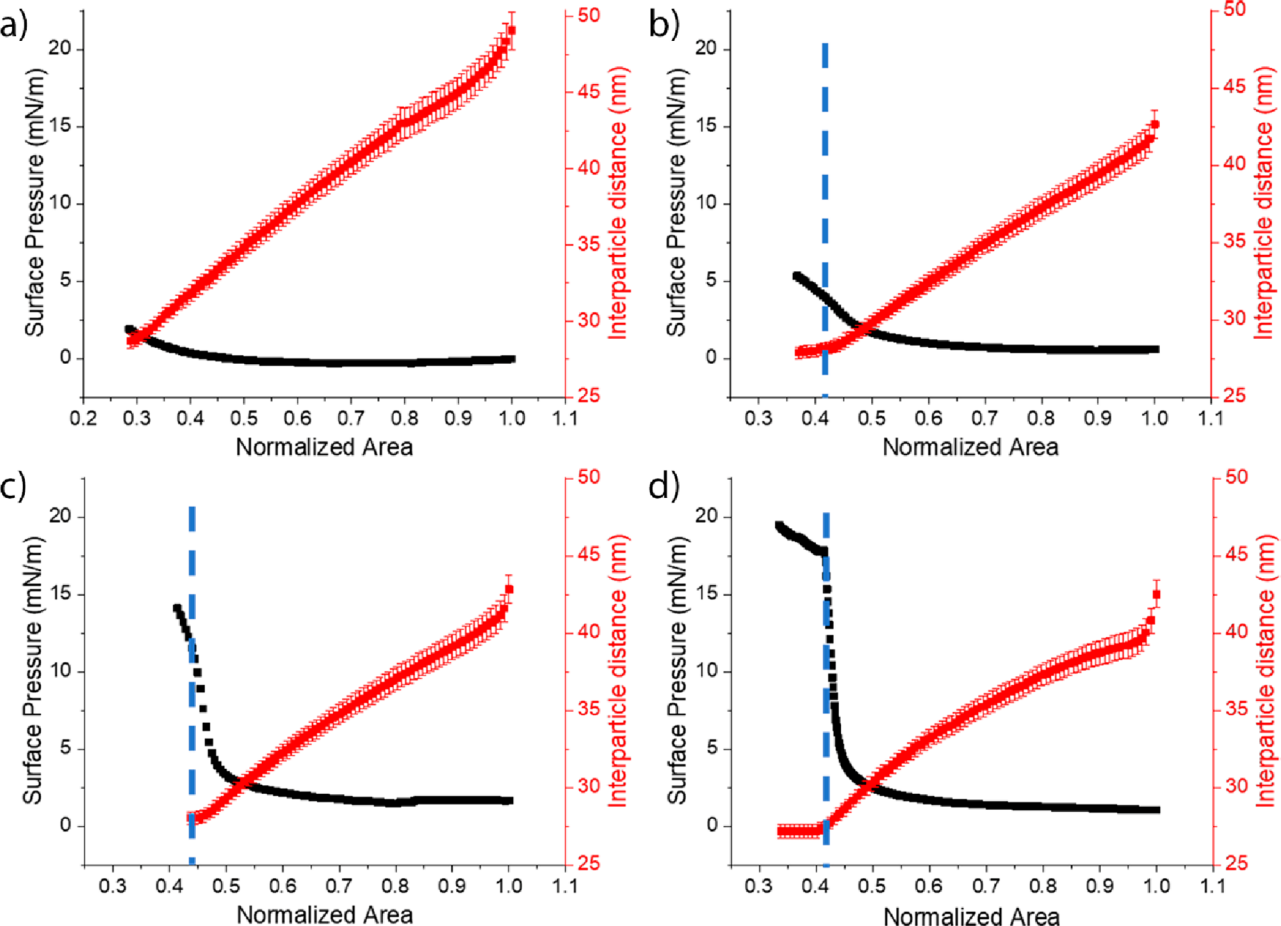
Interparticle distance variation (red) during compression for C_*n*_TAB, 1.1 × 10^–6^ M NaCl, 1 mM NPs 0.1 wt % for the different tail lengths: (a) C_12_TAB, (b) C_14_TAB, (c) C_16_TAB, and (d) C_18_TAB. The black squares are the corresponding surface-pressure values. The dashed lines indicate the limiting compression rate and the corresponding limiting interparticle distance.

**Figure 7:**
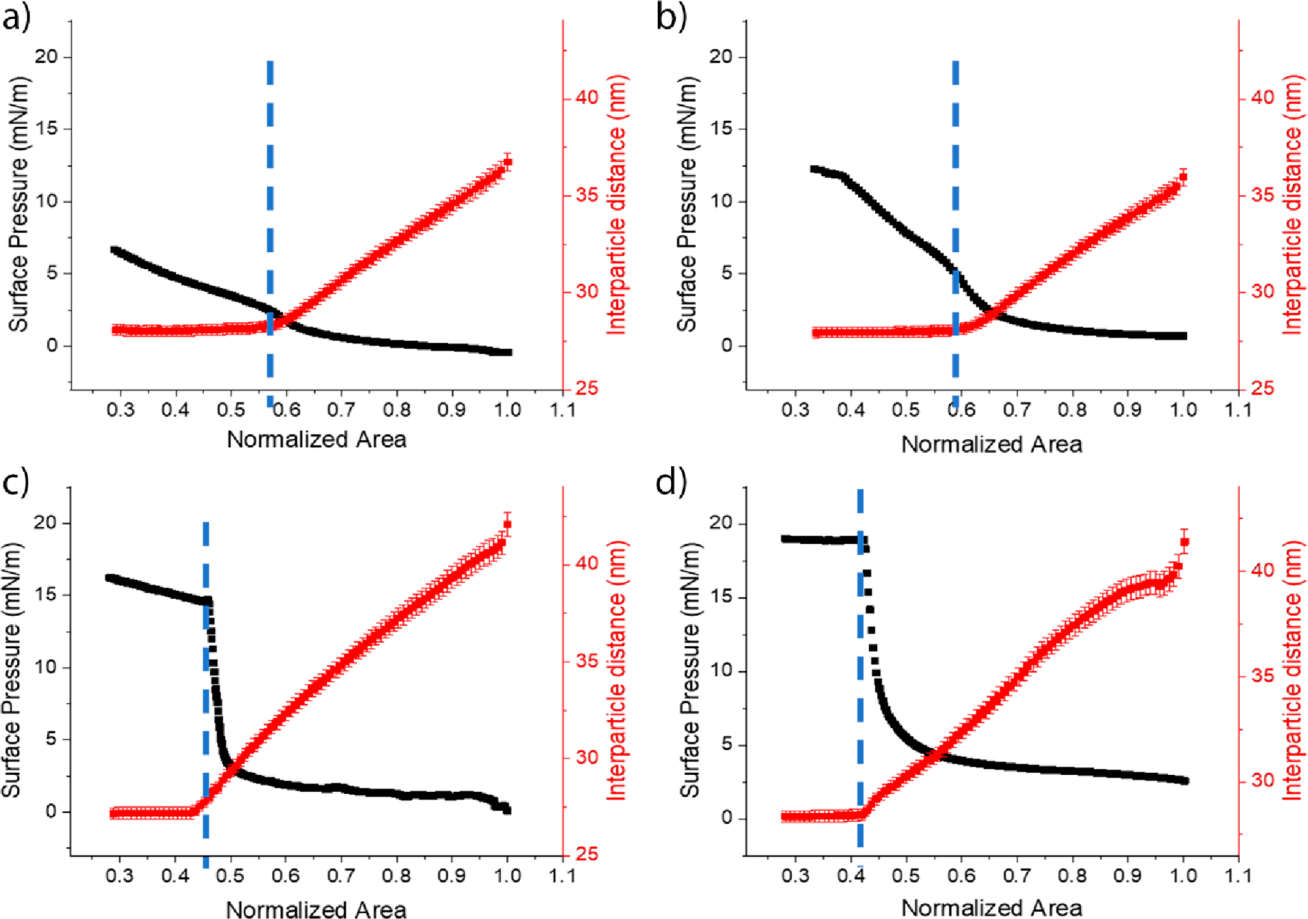
Interparticle distance variation (red) during compression for C_*n*_TAB, 4.4 × 10^–6^ M NaCl, 1 mM NPs 0.1 wt % for the different tail lengths: (a) C_12_TAB, (b) C_14_TAB, (c) C_16_TAB, (d) C_18_TAB. The black squares are the corresponding surface-pressure values. The dashed lines indicate the limiting compression rate and the corresponding limiting interparticle distance.

Further insight into the compression-induced NP reorganization and the related interactions can be obtained from the GISAXS structural characterization reported in Figures 6 and 7, which shows the gradual decrease in the interparticle distance with compression. At both investigated concentrations, the NP monolayers formed with C_18_TAB show steeper interparticle distance reductions in the very early stage of compression, probably because of the residual NP adsorption. The structural characterization allows us to more clearly identify the limiting compression rate of the monolayer as the region where the compression does not induce any further reduction of the interparticle distance except for the NP desorption.^17^ Remarkably, the corresponding limiting interparticle distance does not depend on the surfactant composition or on its concentration, and it is not reached only for the monolayer formed with 1 × 10^–6^ M C_12_TAB. Moreover, the limiting lateral separation between adjacent nanoparticles, ∼3.5 nm, is significantly lower than the length of two stretched C_*n*_TAB surfactant molecules, characterized by the length of the head equal to 0.6 nm^43^ and the tail length equal to 0.15 + 0.1265 × *n* nm.^44^ This suggests that the hydrophobic tails of surfactants adsorbed on adjacent nanoparticles interpenetrate when close enough. At higher surfactant/NP bulk ratios, Maestro et al. observed that exceeding the limiting compression rate led to buckling of the monolayer rather than NP desorption^45^ and that, similar to our case, the limiting compression rate did not depend on the surfactant bulk concentration.^46^ The difference might be due to the increased NP desorption energy with surfactant bulk concentration and to the irreversible compression-induced aggregation in the presence of a larger number of adsorbed surfactant molecules.

**Figure 8:**
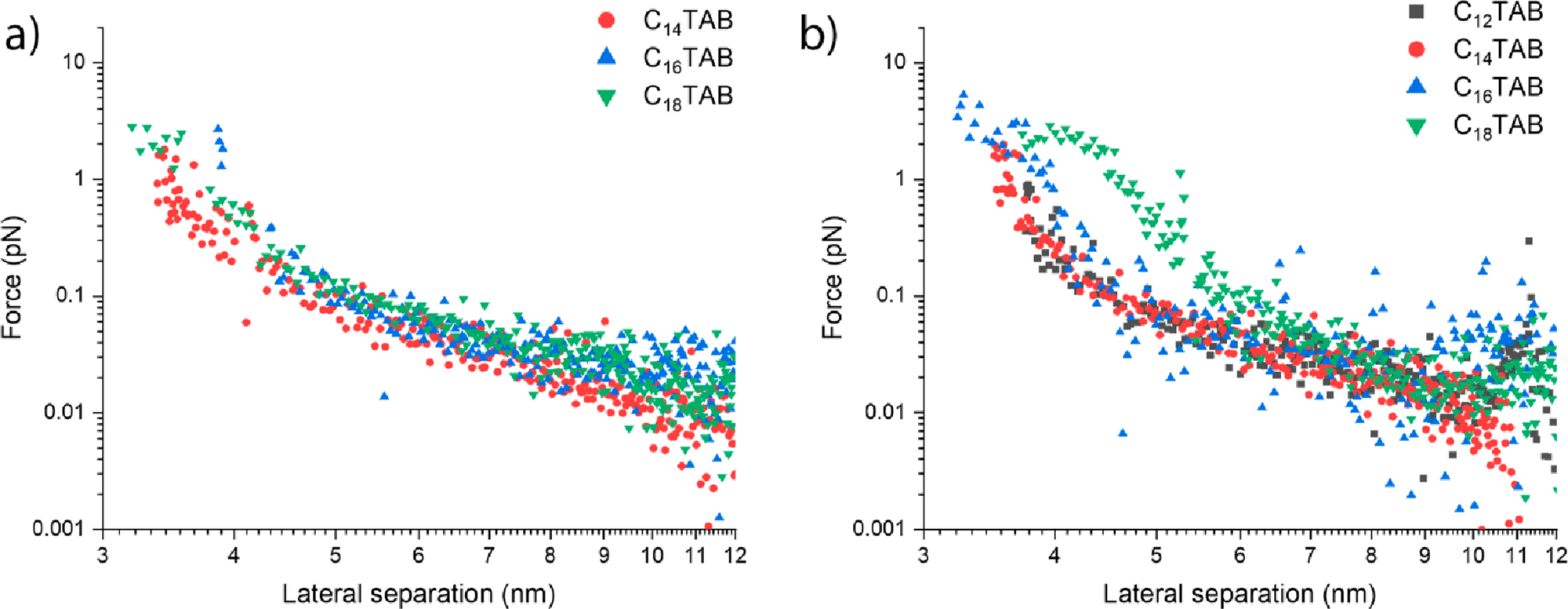
Interfacial interparticle forces calculated from combined GISAXS and compression measurements for the different surfactants (a) C_*n*_TAB, 1.1 × 10^–6^ M NaCl, 1 mM NP 0.1 wt % and (b) C_*n*_TAB, 4.4 × 10^–6^ M NaCl ,1 mM NP 0.1% wt as a function of the lateral separation between adjacent particles.

Thanks to the determination of the interparticle distance, it is also possible to count the number of interfacial nanoparticles during the compression. As is evident (Supporting Information Figures S9 and S10), the number of interfacial NPs decreases steeply only after having reached the limiting interparticle distance, and for normalized areas lower than 0.8, the compression does not induce the NP desorption. The remarkable exception of the C_12_TAB 1.1 × 10^–6^ M monolayer (Figure S9a) is observed, which is presumably related to the lower desorption energy of the corresponding interfacial NPs.^47^ For the other monolayers, between a 0.8 normalized area and the limiting compression rate, the compression work, equal to
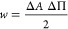
7where Δ*A* is the area reduction and ΔΠ is the surface-pressure increase, is mostly done to approach nanoparticles. It is possible to measure the overall compression work for each portion of the isotherm, and as the number of adsorbed nanoparticles is known, it is possible to measure the work done to reduce the interparticle distance between a given particle and its nearest neighbors. This provides an opportunity to determine, for small interparticle distance reductions, *dD*, and the assumption of hexagonal packing, the repulsive interparticle forces opposing the compression as
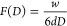
8The so-obtained forces are reported as a function of the lateral separation in Figure 8, showing that the interparticle repulsions extend for about 2 orders of magnitude and depend on the interparticle distance. From these graphs, a general trend, which applies for all surfactants at both concentrations, can be observed.

In particular, two distinct regimes acting, respectively, at long and short range occur, and the transition from one to another is revealed by the change in the slope of the force vs the lateral separation curve. At long range, between approximately 10 and 4 to 5 nm, interparticle forces on the order of 10^–2^ pN occur. These forces scale as ∼*L*^–3^ (Figure S11) and do not depend markedly on the surfactant composition or concentration. This behavior suggests that the main contribution to long-range forces is electrostatic and is not significantly influenced by the presence of surfactants. This suggest that, in the micromolar regime investigated here, the charge of interfacial nanoparticles does not change with surfactant concentration, as already suggested by Anyfantakis et al.,^37^ or tail length. Moreover, the observed independence of long-range forces from the surfactant concentration and structure provides an indirect confirmation of the very small number of interfacial free surfactant molecules. In fact, if surfactants molecules had significantly adsorbed at the interface, they would have screened the electrostatic repulsions among nanoparticles and the strength of the forces would have varied accordingly, as more concentrated and longer-tailed surfactants would lead to a more pronounced adsorption and thus to a more effective screening.

The observed scaling with lateral separation of long-range forces is also close to values previously reported in both experimental and theoretical studies. In particular, for charged surfactant-free microparticles at the air–water interface, the repulsions scale as *L*^–4^(13,48) and originate from the formation of interfacial dipoles.^49^ The scaling predicted for electrostatic charge–charge and charge–image charge interactions at the interface between a dielectric and an electrolyte solution is *L*^–2^.^50^ Indeed, the deviation from the commonly observed *L*^–4^ dependence may suggest a non-negligible effect of image charges^48^ due to either nonexclusive dipolar repulsion or to the low interparticle distance/NP radius ratios, lower than the ones reported in the literature for interfacial microparticles.^13,48^ Further investigations are currently ongoing in order to extract more detailed information about the origin of long-range repulsions. Finally, although adsorbed surfactant/NP complexes may undergo significant reorganization leading to structure and stoichiometry which may significantly diverge from the bulk values,^31^ it is worth observing that, at surfactant/NP bulk ratios comparable to the ones here investigated (10–40), the NP bulk charge is also unaffected by the surfactant tail length and the concentration.^16^

On the other hand, the behavior is different for short-range interactions, where force values and scaling depend on the surfactant concentration and nature. This is particularly evident for the C_18_TAB surfactant, displaying higher forces that also change their extent and scaling with concentration (Supporting Information Figure S13). This evidence, together with the trend in compression isotherms, proves the hypothesis of short-range ligand-mediated forces dominated by the steric repulsions, which imply the adsorption of surfactant molecules onto the interfacial nanoparticles. The adsorption of a different number of molecules with concentration leads, for the C_18_TAB surfactant, to a change in the lateral separation range where short-range repulsions act. In particular, while at 1 × 10^–6^ M steric forces act at lateral separations lower than 4 nm, at 4.4 × 10^–6^ M the range is extended to up to 5.5 nm. This behavior can be interpreted by considering that the larger number of molecules adsorbed on each nanoparticle reduces the conformational flexibility of the tails, which adopt a more stretched conformation leading to a longer range of action for steric repulsions. The increased highest distance of short-range repulsions with C_18_TAB concentration suggests that immersion capillary attractions, which can be higher than *k*_B_*T* even for NPs,^51^ are not relevant for the system investigated here because their strength should increase with the NP hydrophobicity, i.e., with surfactant concentration. Remarkably, the short-range force dependence on concentration is weaker or even negligible for surfactants with shorter tails (Supporting Information Figure S13). This evidence, which is in agreement with the behavior shown by the compression moduli, suggests that although micromolar numbers of cationic ligands already cause the onset of short-range steric repulsions, the number of surfactant molecules decorating each NP is not high enough to significantly vary the strength and the lateral separation range of steric repulsions when the shortest tails are involved. The low decoration number was already suggested by two other pieces of evidence: the limiting lateral separation, which is significantly shorter than the length of two surfactant molecules and the independence of long-range electrostatic repulsions from the surfactant concentration, proving that the number of decorating molecules is not high enough to reduce the NP surface charge. This low decoration number leads to low NP interfacial contact angles^31^ and, therefore, to the predominant immersion of interfacial NPs in the aqueous phase. Therefore, the few surfactant molecules attached to interfacial NPs and exposed to the air phase are sufficient to generate steric repulsions, although they do not significantly reduce the nanoparticle charge.

Finally, it is worth observing how, at 4.4 × 10^–6^ M surfactant concentration, the slope of the short-range forces decreases with increasing the tail length (Figure 8b), from 14.2 ± 5.7 for C_12_TAB to 8.5 ± 1.2 for C_18_TAB (Supporting Information Figure S12). Although at a given lateral separation steric forces are higher for longer tails, the increase is more rapid for shorter ones. The compression isotherms of C_18_TAB-only solutions suggested the presence of slowly desorbing free surfactant molecules (see Supporting Information Figures S7 and S8 and the previous discussion). However, any contribution to surface pressure arising from interfacial free surfactant molecules would rather be additive and, in turn, would lead to steeper changes in interparticle forces with surfactant concentration. As this is not the case, we believe that the reduced slope cannot be accounted for by the presence of residual adsorbed free C_18_TAB molecules. On the contrary, this behavior, which was already observed for polymer-grafted particles,^52^ is attributable to the higher flexibility of longer tails that, when compressed, adopt coiled conformations characterized by an entropic gain proportional to the tail length. This entropy-driven flexibility provides another confirmation of the steric nature of short-range repulsions and therefore demonstrates that only a few surfactant molecules decorate each interfacial nanoparticle. It is reasonable to expect that the distribution of surfactant molecules onto interfacial nanoparticles is not homogeneous and tends to minimize the surface free energy by exposing the tails to air. Given the high residual charge of nanoparticles^16^ and therefore the very low contact angle, the portion exposed to the air is likely a small fraction of the whole NP surface. Although the number of surfactant molecules decorating each adsorbed NP cannot be determined, the argument reported above suggests that this number is small. Nevertheless, both the monolayer structure and compressibility are significantly influenced by the surfactant nature and concentration, leading to the possibility of an ultrafine modulation of the monolayer properties by careful formulation of the dispersion composition. On the other hand, when interfacial nanoparticles are decorated with a larger number of surfactants, other interparticle interactions, including hydrophobic attractions,^45,46^ might also occur.

## Conclusions

Our results open the door to the comprehensive investigation and comprehension of picoforces acting between nanoscale objects confined at a liquid interface over a broad range of interparticle distances. Our approach, which allows us to investigate how surfactants influence both the magnitude of repulsive forces and the interparticle distance range where surfactant-mediated forces act, showed that interparticle forces are influenced even by the addition of micromolar quantities of cationic surfactants. In particular, we identified two main contributions to repulsions. Long-range forces, which are not affected by the surfactant structure and concentration, have mainly an electrostatic origin. Short-range interparticle forces are related to the hydrophobic tail length and surfactant concentration, and their scaling with distance and tail length is consistent with a predominant steric contribution. The steric nature of short-range forces also demonstrates that, even in the micromolar regime, cationic surfactants decorate negatively charged nanoparticles thus prompting their adsorption at the air/water interface. Our force characterization is also the first experimental report of the entropy-driven relaxation of short hydrophobic chains at the air/water interface. We believe that our approach can be applied to a vast range of interfacial systems, thus providing a powerful tool for the comprehension of forces acting between nanoscale objects and for the fine modulation of 2D and quasi-2D nanostructures. Moreover, interfacial nanoparticles can be employed as carriers of surfactants, thus enabling the measurement with subnanometer resolution of repulsions acting among various surface-active molecules.

## Supporting Information Available

TEM characterization of nanoparticles, synchrotron-radiation damage determination, GISAXS fits of static structural characterizations, compression isotherms of surfactant solutions and surfactant/NP dispersion, evolution of the number of interfacial NPs during the compression, fits of long-range interparticle forces and short-range interparticle forces, and a comparison of interparticle forces as a function of the surfactant bulk concentration (PDF)
