# Supplementary material for: Direct Measurement of Surfactant-Mediated Picoforces among Nanoparticles in a Quasi-Two-Dimensional Environment
Source: Langmuir. 2022 Sep 29;38(40):12281–91. doi: 10.1021/acs.langmuir.2c01928 (PMC9558483; doi:10.1021/acs.langmuir.2c01928)
Supplement: Supplementary file 1 — la2c01928_si_001.pdf [file la2c01928_si_001.pdf]

SUPPORTING INFORMATION

Direct measurement of surfactant-mediated pico-forces among nanoparticles in a quasi-2D environment

Roberta Ruffino[a], Nunzio Tuccitto[a], Gianfranco Sfuncia[b], Giuseppe Nicotra[b], Giovanni Li-Destri[a], Giovanni Marletta[a]*

[a] Laboratory for Molecular Surfaces and Nanotechnology (LAMSUN) and CSGI, Department of Chemical Sciences, University of Catania, viale A. Doria 6, 95125, Catania, Italy.

[b] Consiglio Nazionale delle Ricerche, Istituto per la Microelettronica e Microsistemi, Catania I, 95121, Italy

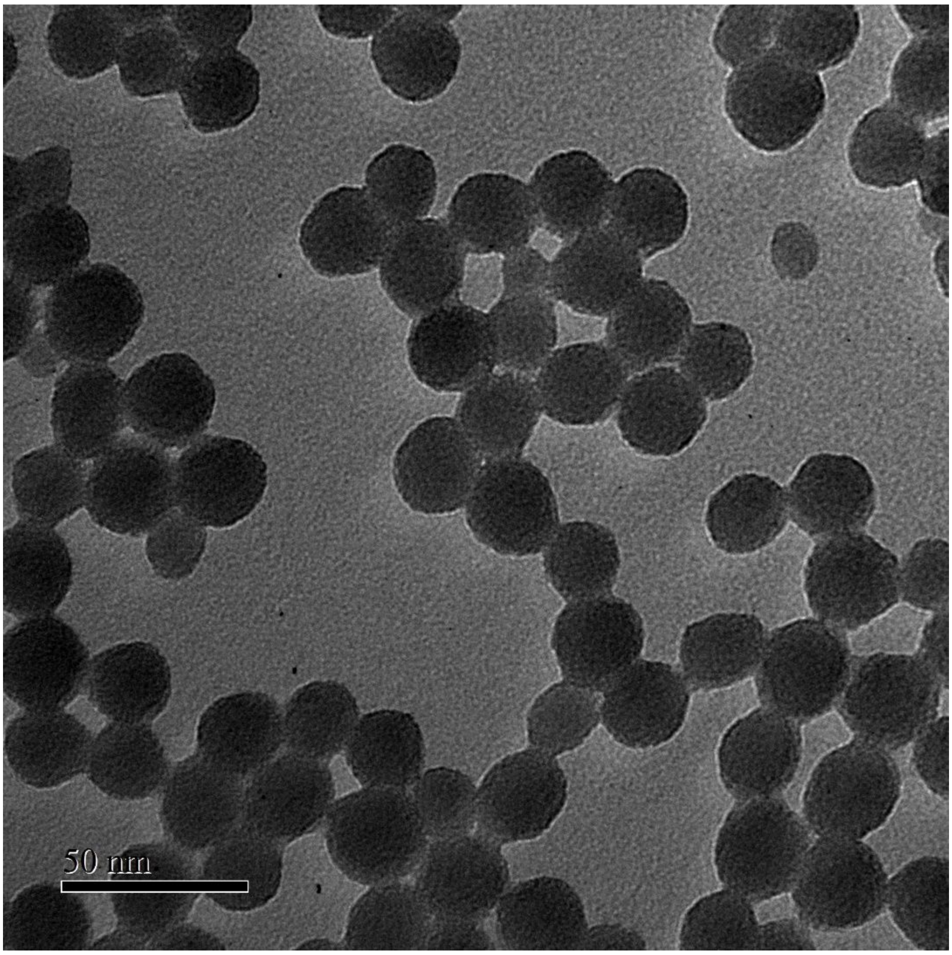

Figure S1: TEM micrographs of silica nanoparticles.

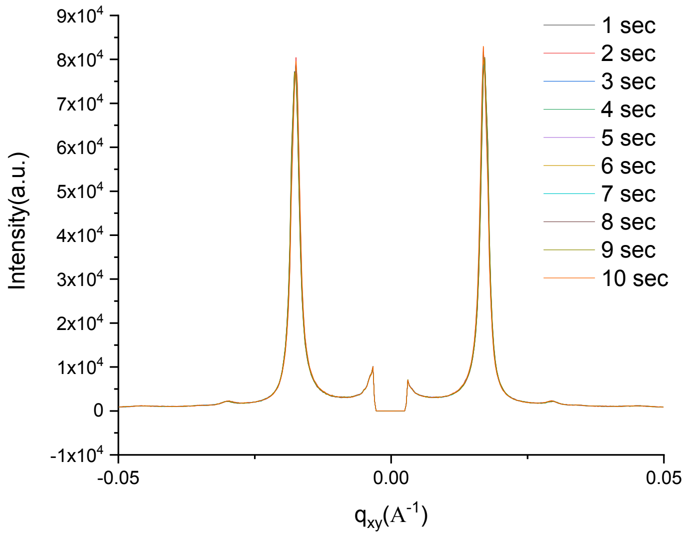

Figure S2: Evolution of the GISAXS structure factor with illumination. After ten subsequent 1-second illuminations the peaks shape and intensity is not altered. This demonstrate that the employed experimental conditions allow avoiding any radiation damages.

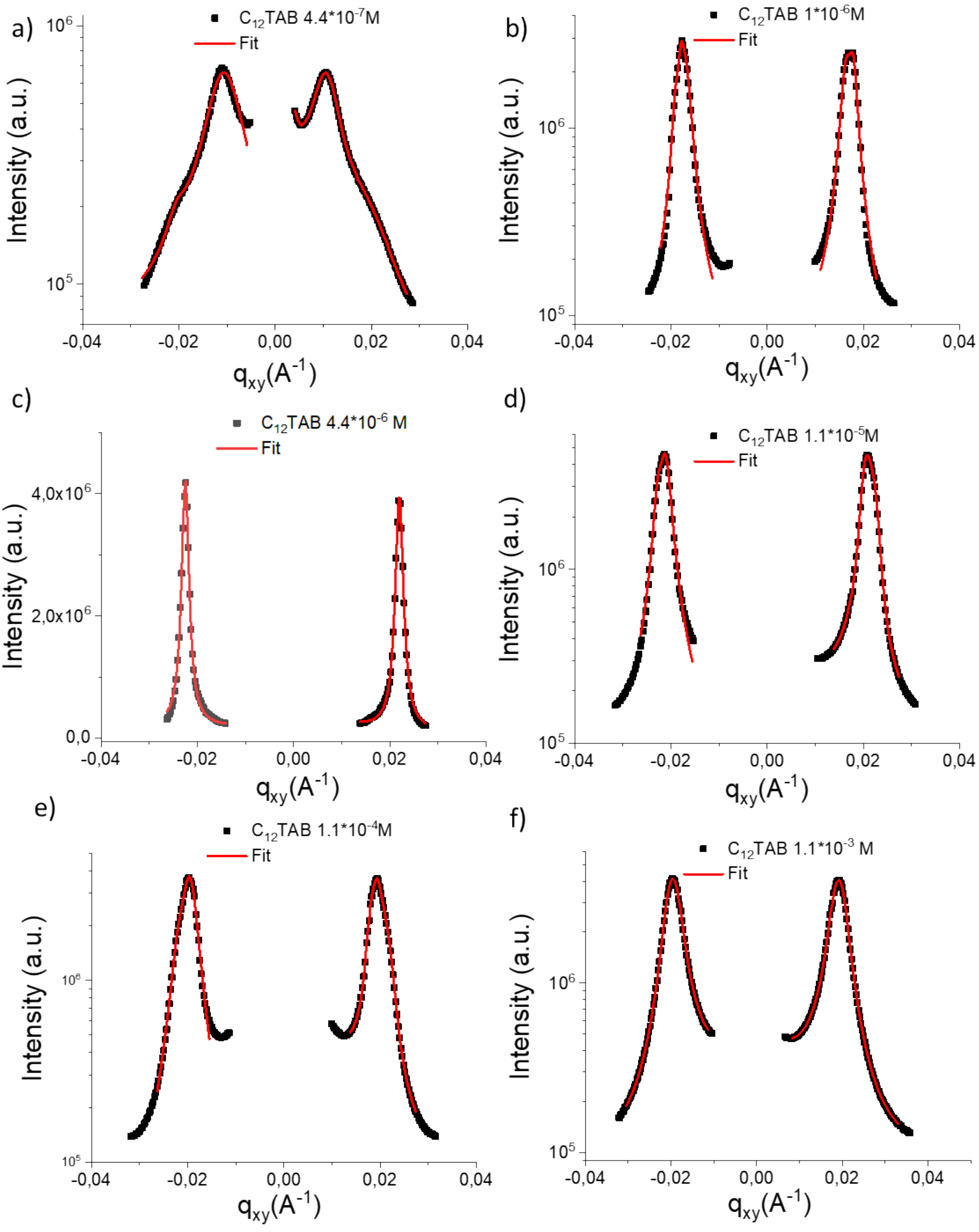

Figure S3: GISAXS cuts (black) and fit (red) of monolayers formed from dispersions having NaCl 1mM, NPs 0.1% wt and $C_{12}TAB$ 4.4*10^{-7}M (a), 1.1*10^{-6}M (b), 4.4*10^{-6}M (c), 1.1*10^{-5} M (d), 1.1*10^{-4}M (e), 1.1*10^{-3}M (f).

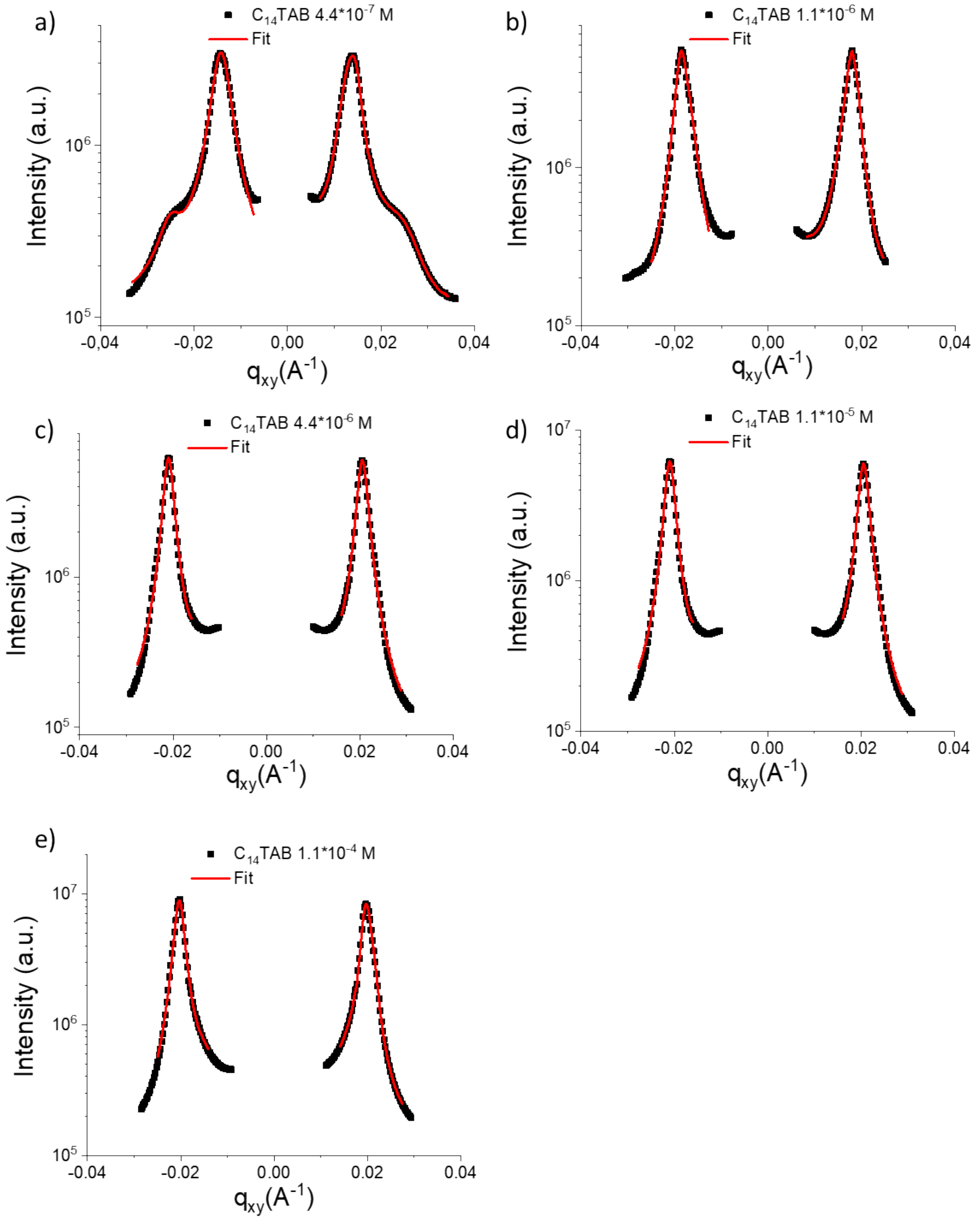

Figure S4: GISAXS cuts (black) and fit (red) of monolayers formed from dispersions having NaCl 1mM, NPs 0.1% wt and C_{14}TAB $4.4*10_{-7}$M (a), $1.1*10^{-6}$M (b), $4.4*10^{-6}$M (c), $1.1*10^{-5}$ M (d) $1.1*10^{-4}$M (e).

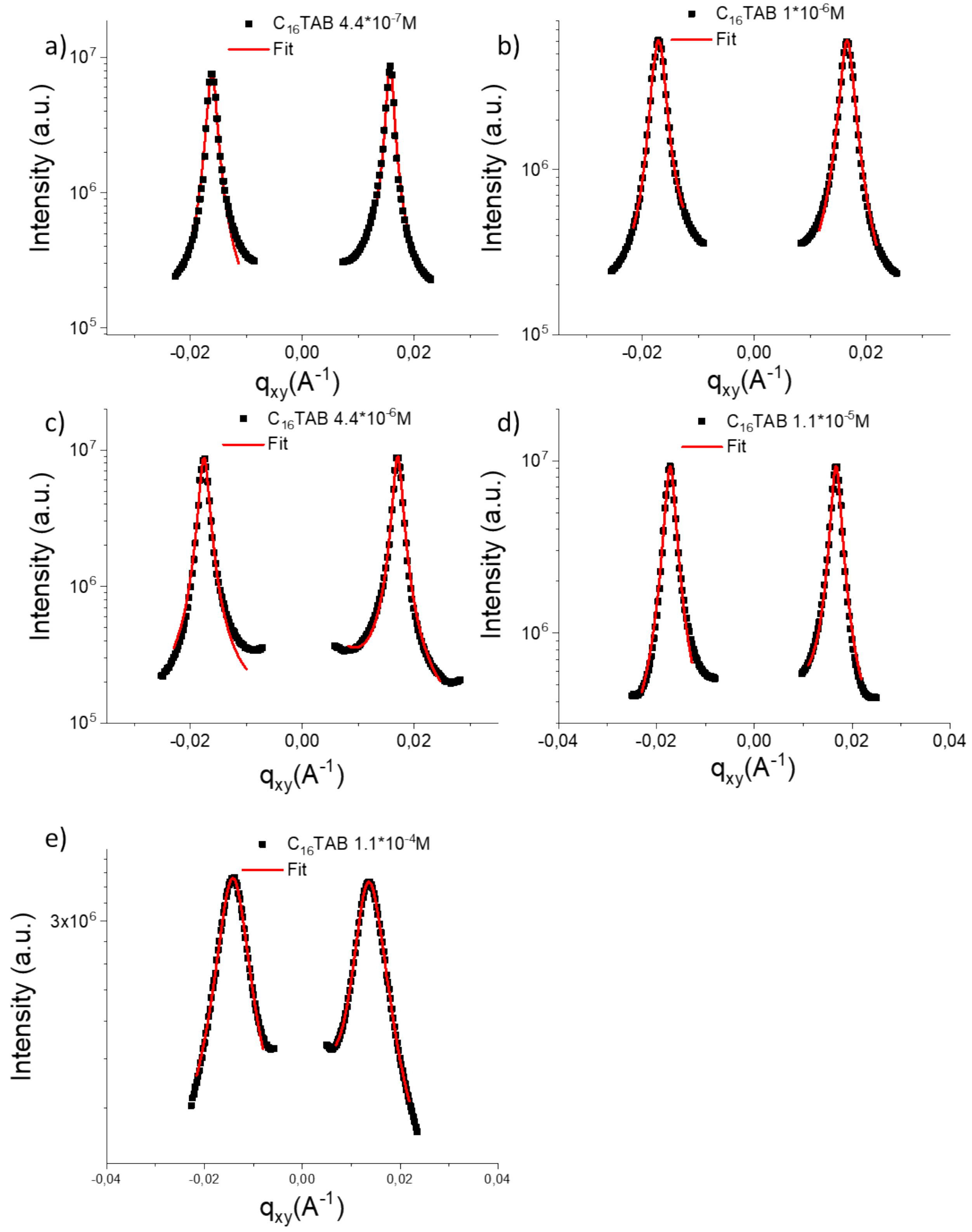

Figure S5: GISAXS cuts (black) and fit (red) of monolayers formed from dispersions having NaCl 1mM, NPs 0.1% wt and $C_{16}TAB$ 4.4*10^{-7}M (a), 1.1*10^{-6}M (b), 4.4*10^{-6}M (c), 1.1*10^{-5} M (d) 1.1*10^{-4}M (e).

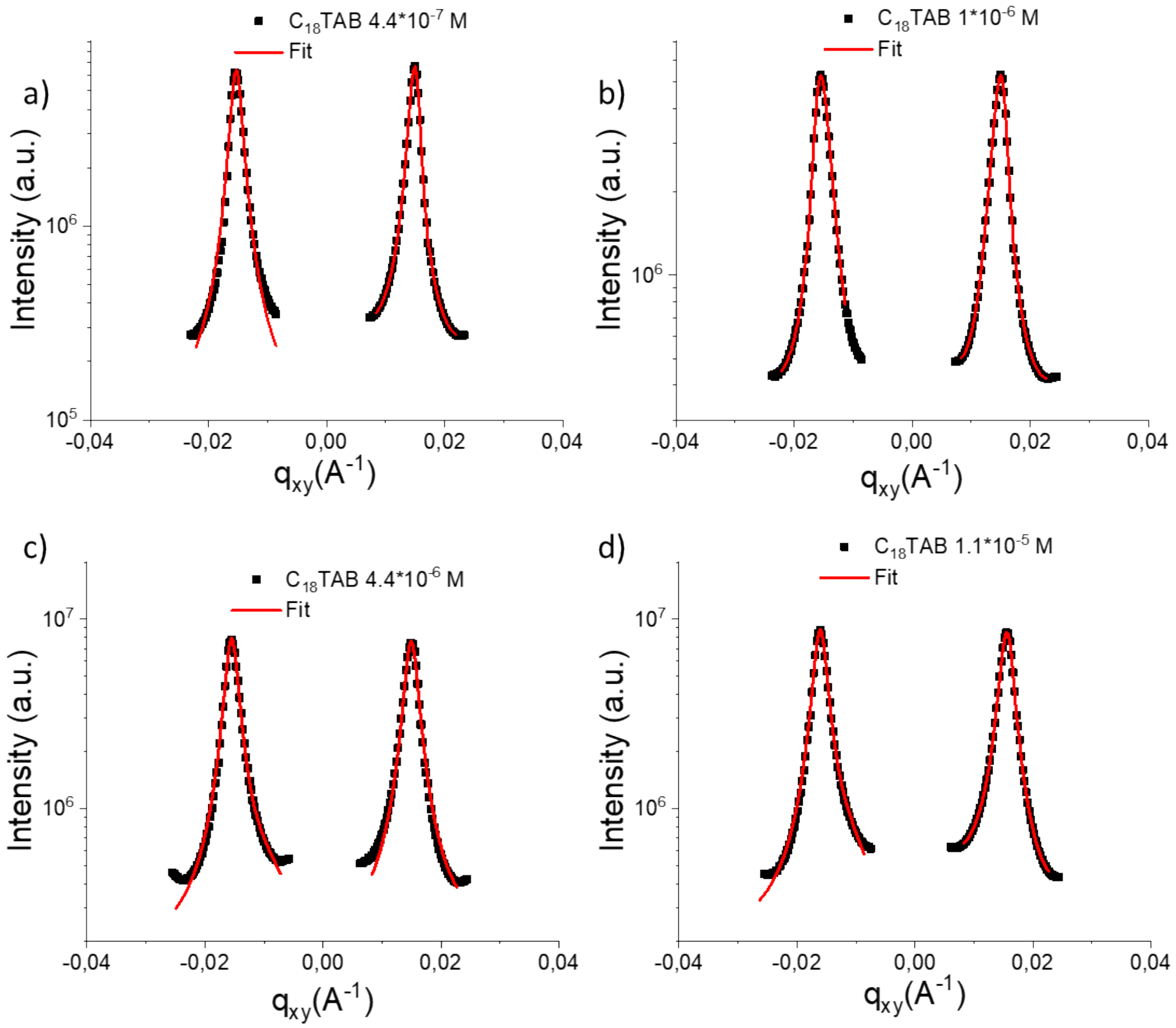

Figure S6: GISAXS cuts (black) and fit (red) of monolayers formed from dispersions having NaCl 1mM, NPs 0.1% wt and $C_{18}TAB$ $4.4*10^{-7}$M (a), $1.1*10^{-6}$M (b), $4.4*10^{-6}$M (c), $1.1*10^{-5}$ M (d) $1.1*10^{-4}$M (e).

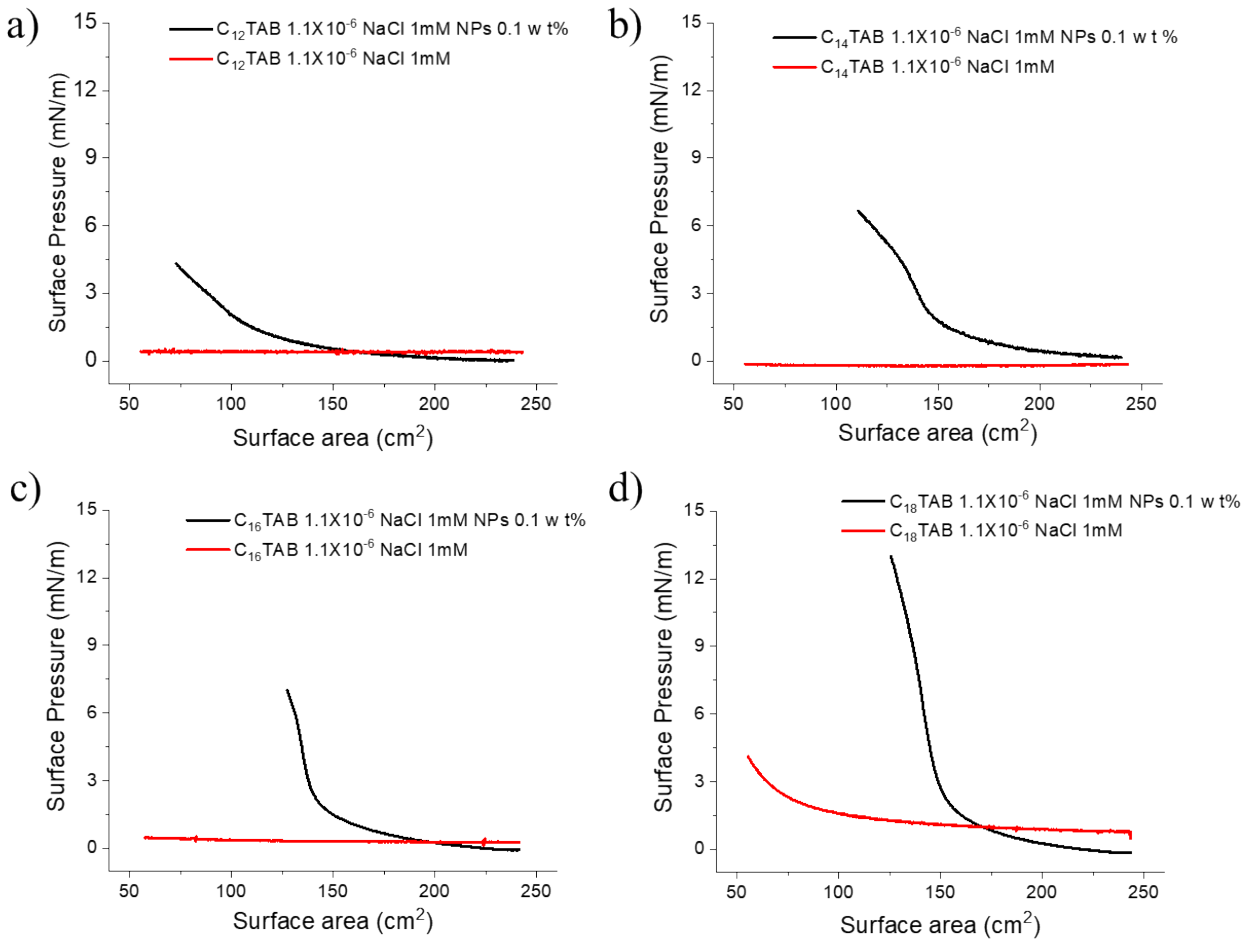

Figure S7: Compression isotherms recorded for dispersions having NaCl 1mM, NPs 0.1% wt and C_nTAB 1.1*10^{-6}M (black) and for solutions having NaCl 1mM and C_nTAB 1.1*10^{-6}M (red): C_{12}TAB (a), C_{14}TAB (b), C_{16}TAB (c), C_{18}TAB (d).

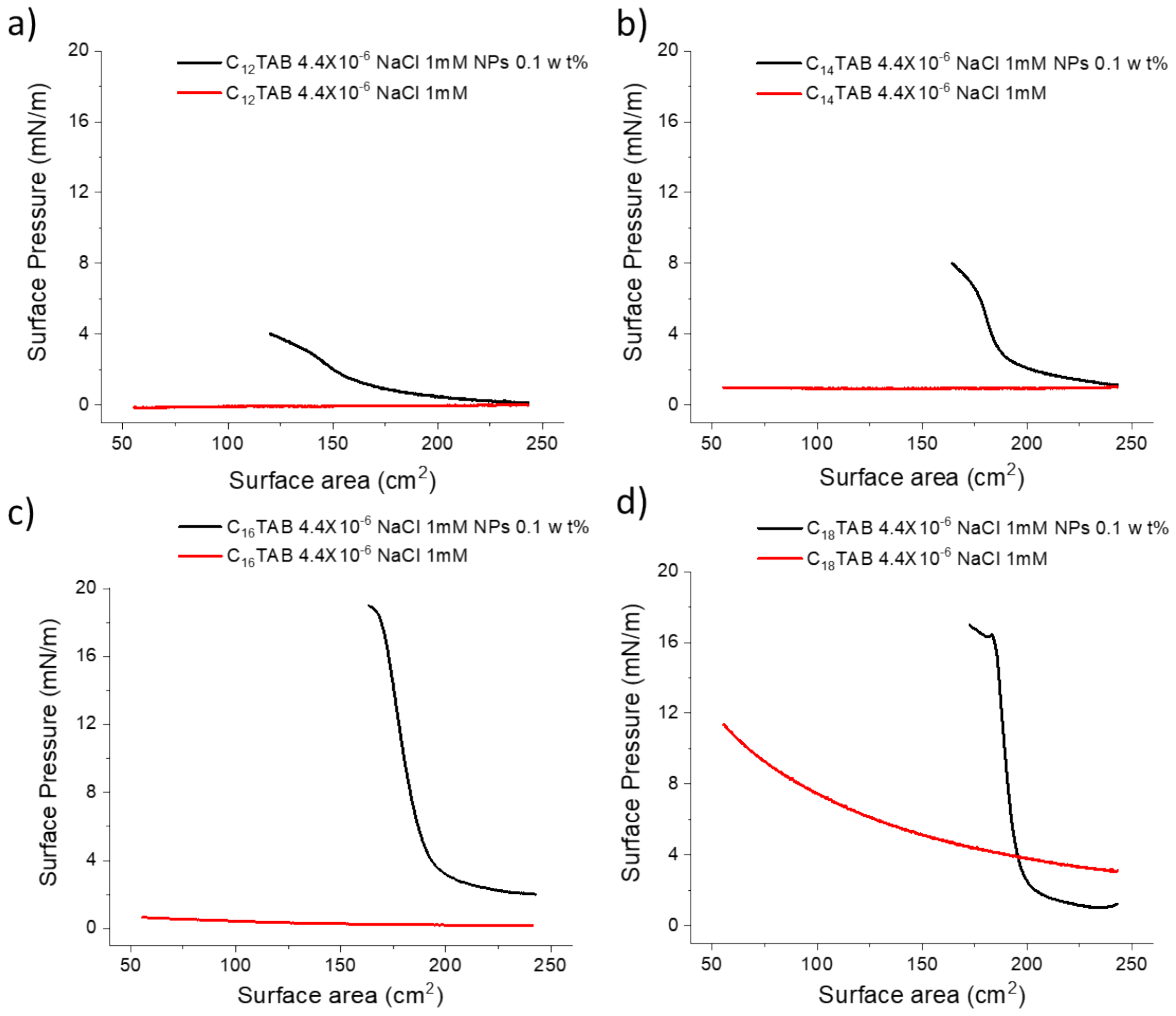

Figure S8: Compression isotherms recorded for dispersions having NaCl 1mM, NPs 0.1% wt and C_nTAB $4.4*10^{-6}M$ (black) and for solutions having NaCl 1mM and C_nTAB $4.4*10^{-6}M$ (red): $C_{12}TAB$ (a), $C_{14}TAB$ (b), $C_{16}TAB$ (c), $C_{18}TAB$ (d).

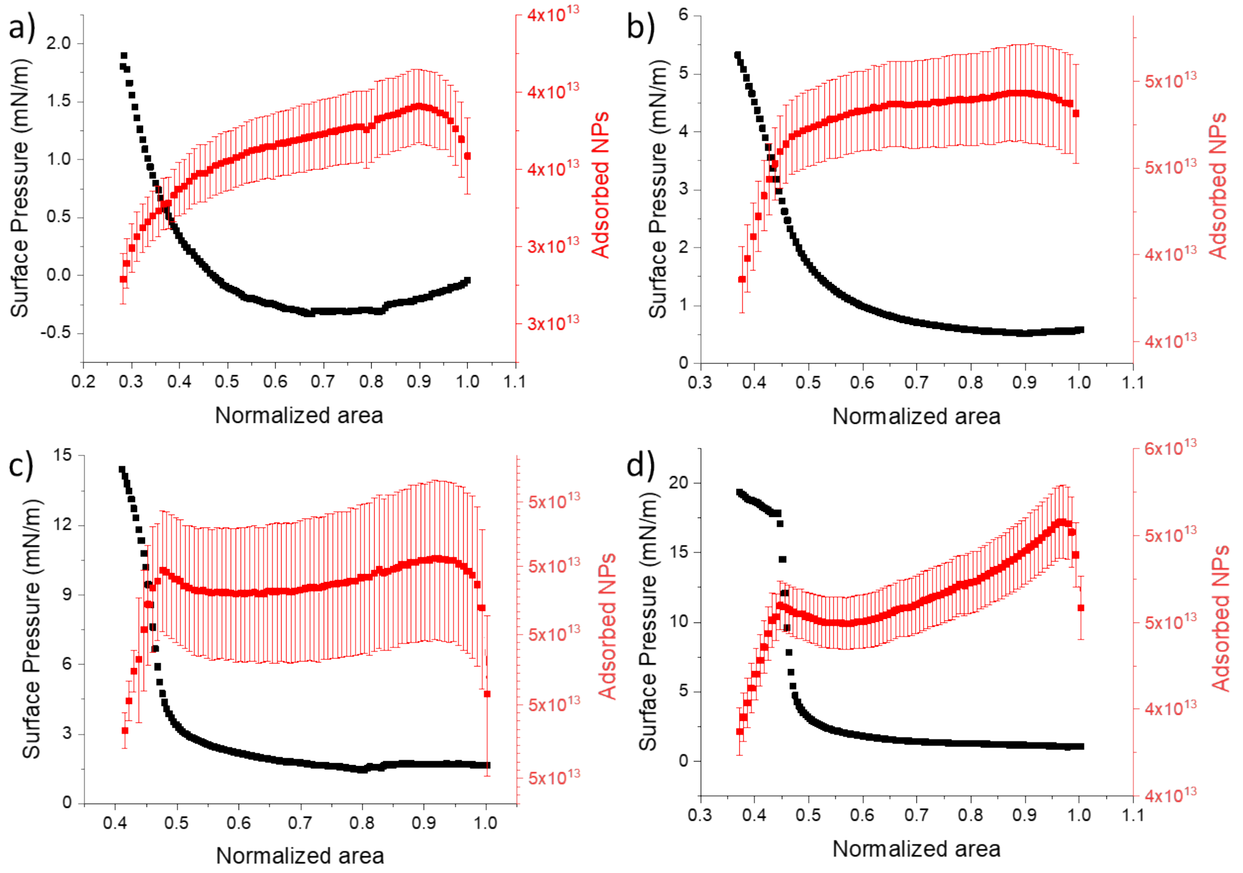

Figure S9: Calculated number of adsorbed particles (red) and the corresponding compression isotherm (black) recorded for dispersions having NaCl 1mM, NPs 0.1% wt and C_nTAB $1.1*10^{-6}$M (black) and for solutions having NaCl 1mM and C_nTAB $1.1*10^{-6}$M (red): $C_{12}TAB$ (a), $C_{14}TAB$ (b), $C_{16}TAB$ (c), $C_{18}TAB$ (d).

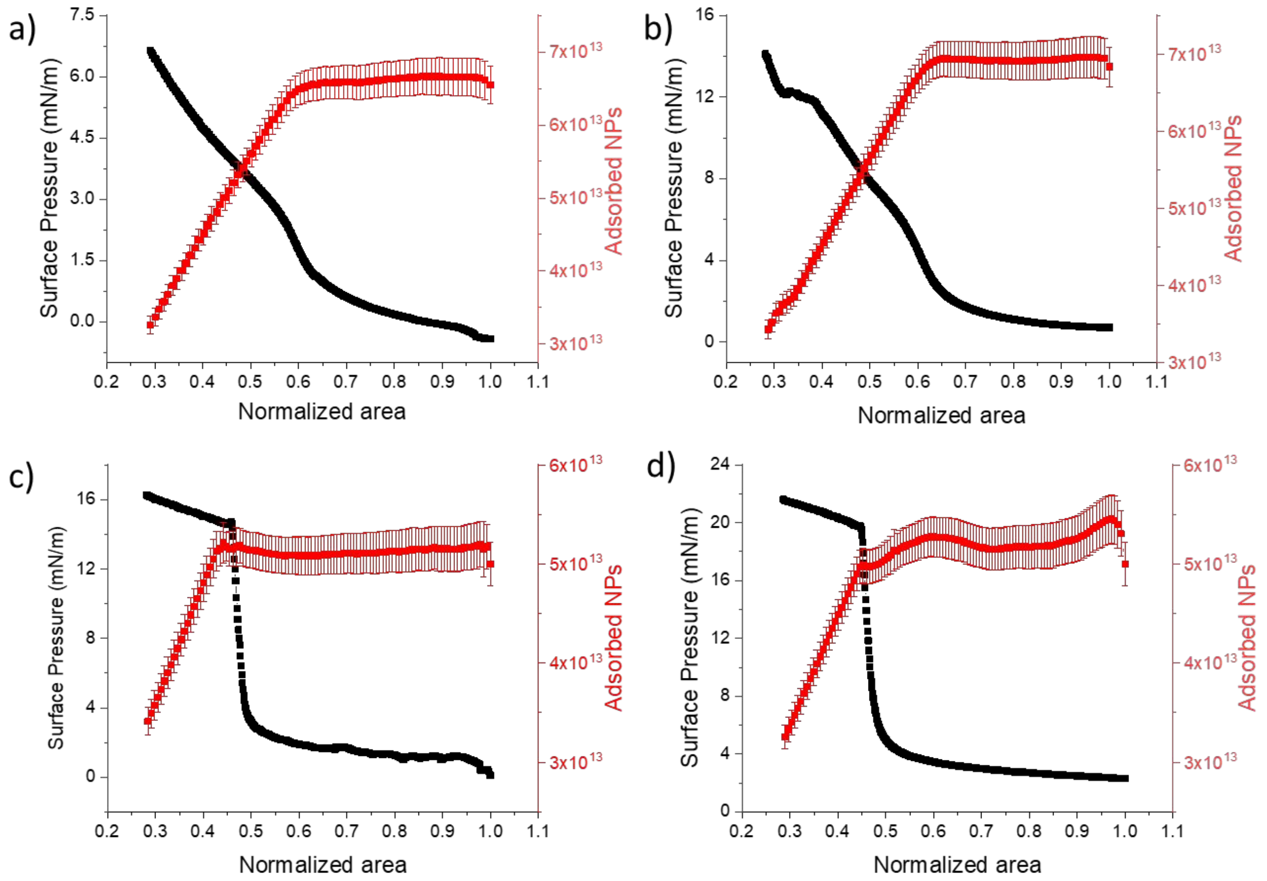

Figure S10: Calculated number of adsorbed particles (red) and the corresponding compression isotherm (black) recorded for dispersions having NaCl 1mM, NPs 0.1% wt and C_nTAB 4.4*10^{-6}M (black) and for solutions having NaCl 1mM and C_nTAB 4.4*10^{-6}M (red): $C_{12}TAB$ (a), $C_{14}TAB$ (b), $C_{16}TAB$ (c), $C_{18}TAB$ (d).

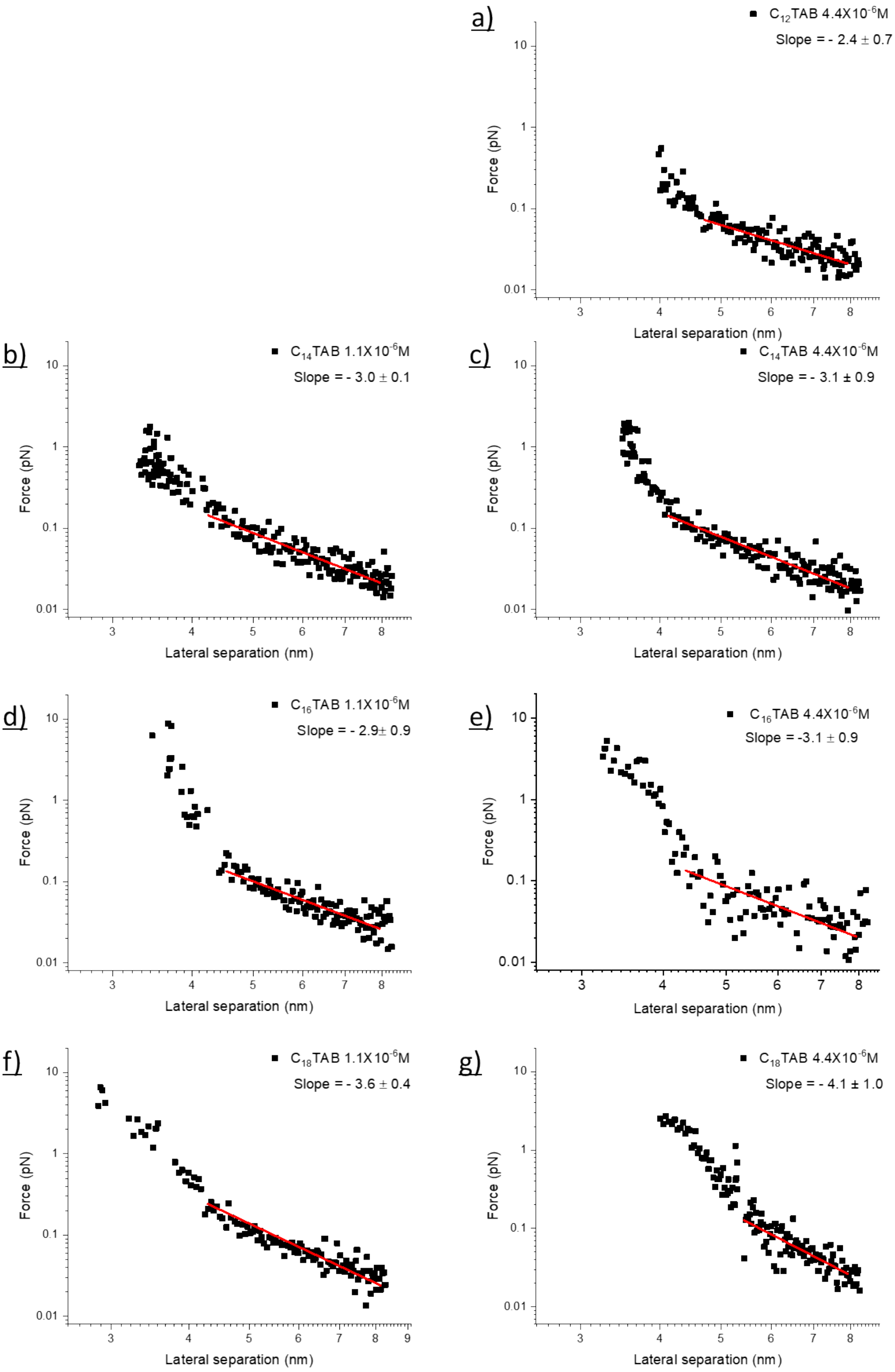

Figure S11: Linear fit (red) of the long-range region of the log-log force plot (black) calculated for NP monolayers formed with surfactants with different chain length and concentration (see text of each plot). The linear fit angular coefficient ranges between 2.2 and 4.1 and it does not show significant variations with surfactant chain length and concentration.

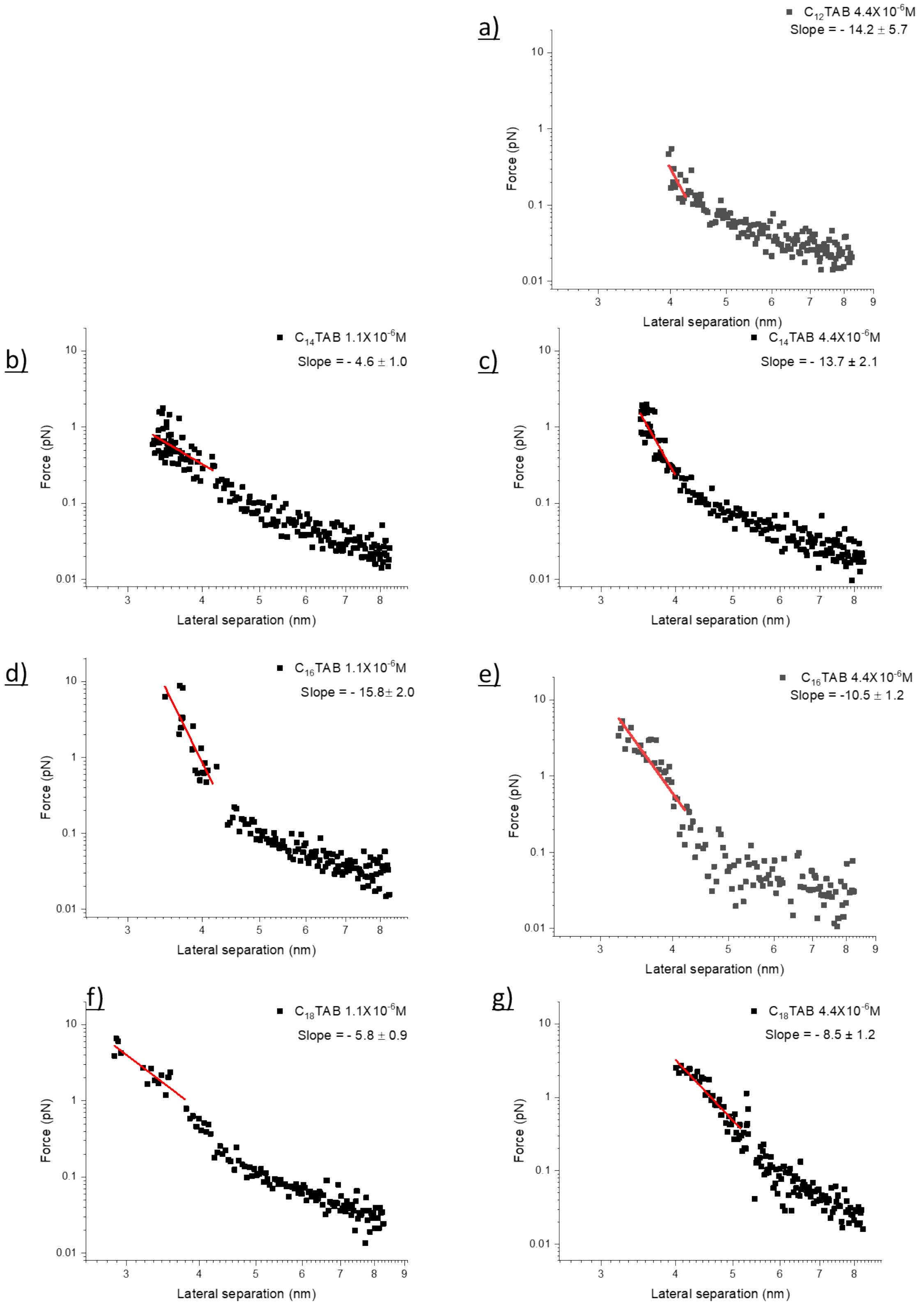

Figure S12: Linear fit (red) of the short-range region of the log-log force plot (black) calculated for NP monolayers formed with surfactants with different chain length and concentration (see text of each plot).

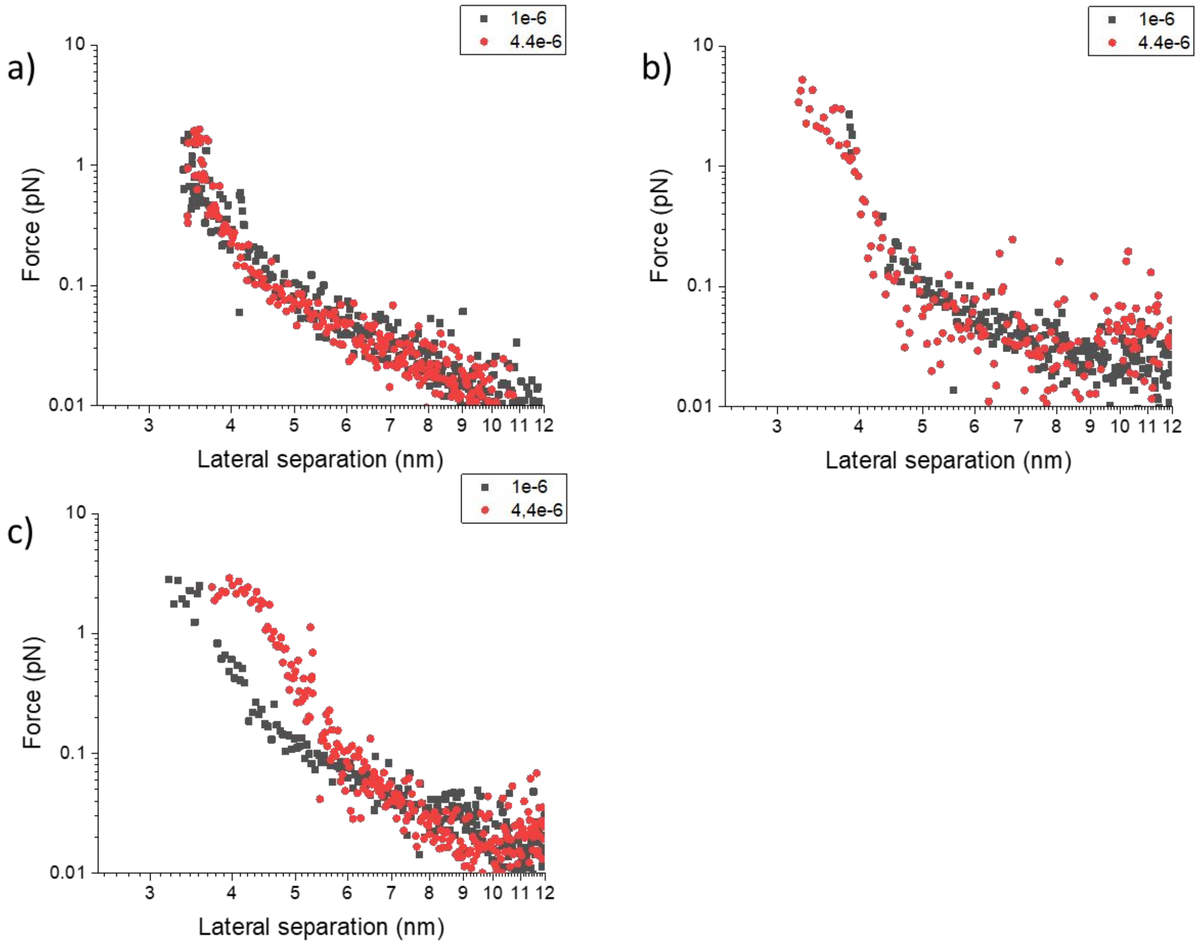

Figure S13: Comparison of inter-particle forces as a function of the lateral separation measured during the compression of monolayers formed from dispersion having NaCl 1mM, NPs 0.1% wt and C_nTAB 1.1*10^{-6}M (black) and NaCl 1mM, NPs 0.1% wt and C_nTAB 4.4*10^{-6}M (red): $C_{14}TAB$ (a), $C_{16}TAB$ (b), $C_{18}TAB$ (c).
